# The plastisphere: not a unique biofilm but a unifying concept for the interdisciplinary plastic biofilm research community

**DOI:** 10.1093/femsec/fiag055

**Published:** 2026-06-22

**Authors:** Priscilla Carrillo-Barragán, Elisenda Ballesté, Michael Sauer, Joseph Christie-Oleza

**Affiliations:** Department of Plant and Microbial Biology, University of Zurich, 8008 Zurich, Switzerland; Departament de Genètica, Microbiologia i Estadística, Universitat de Barcelona, 08028 Barcelona, Spain; OMV Aktiengesellschaft, Headoffice, Trabrennstr. 6-8, 1020 Vienna, Austria; Department of Biology, University of the Balearic Islands, 07122 Palma de Mallorca, Spain

**Keywords:** plastisphere, biofilm ecology, biofilm, microbial dispersal, plastic pollution, microbial community assembly

## Abstract

Plastic has introduced a novel and persistent substrate into natural ecosystems, rapidly colonized by microbial biofilms collectively termed the plastisphere. Since its introduction, the concept has catalyzed interdisciplinary research and shaped scientific and public discourse on plastic pollution. Yet, a central question remains unresolved: Do plastisphere communities represent a fundamentally distinct ecological entity, or are they conventional biofilms forming on an unconventional material? Here, we synthesize current evidence across marine and terrestrial systems to argue that plastisphere communities are not consistently taxonomically or functionally unique. Instead, they largely reflect established biofilm assembly processes governed by environmental conditions, source communities, and successional dynamics. Claims of plastic biodegradation, pathogen enrichment, or antimicrobial resistance hotspots remain context-dependent and often lack robust comparative frameworks. We propose that the ecological significance of the plastisphere lies not in microbial novelty, but in the properties of the substrate itself. Plastics are uniquely persistent and, in many environments, highly mobile, enabling microbial communities to disperse across ecosystems and extend residence times beyond those of natural particles. By reframing the plastisphere as a condition of microbial life on durable, mobile substrates, we retain its conceptual value while aligning it with ecological theory and advancing a more precise research agenda.

## Teaser

Microbial communities on plastics are not fundamentally different from other biofilms, yet plastics create long-lasting, mobile habitats that change how microbes spread and connect ecosystems.

## Introduction

Since its introduction in the landmark paper by Amaral-Zettler et al. (2013), the term plastisphere has become a shorthand for the microbial communities colonizing plastic debris in aquatic and terrestrial systems. Its value has been clear: The term attracted attention, framed an emerging research agenda, increased the eco-social awareness of plastic pollution, and resonated with the public and policymakers alike (Amaral-Zettler et al. 2020).

More than a decade later, however, it is worth reflecting on what the term implies. Are plastic-associated communities ecologically distinct from other biofilms, or are we just describing common biofilms on a novel substrate? As research has matured, evidence suggests that the answer lies somewhere in between.

In this opinion piece, we argue that bulk plastispheres do not consistently denote taxonomic novelty or guaranteed metabolic exceptionalism. Rather, their ecological relevance stems from the persistence and mobility of synthetic polymers in the environment. By clarifying this distinction, we aim to retain the conceptual usefulness of the term without overstating its biological uniqueness. At the same time, we recognize that substrate–specific interactions may still occur at smaller spatial scales or during early colonization stages, before many mature plastisphere communities converge towards more typical biofilms. We therefore encourage the research community to move beyond bulk plastisphere analyses and investigate the structural, spatial, and functional organization of these biofilms, particularly at the plastic–microbe interface.

## Plastic polymers as microbial substrates

Before evaluating whether the plastisphere represents a distinct ecological niche, it is first necessary to consider the nature of the substrate itself: plastic polymers. Plastics are not a single material but a broad class of synthetic polymers encompassing diverse chemical structures, physicochemical properties, and biodegradability (Obrador-Viel et al. 2024).

Currently, biodegradable synthetic polymers account only for ∼0.3% of global plastic production and, indeed, may in some cases select for distinct microbial communities (Dussud et al. 2018, Gambardella et al. 2025) as would occur in biofilms found on other natural biodegradable materials. However, this is not always the case. For instance, polylactic acid (PLA) shows very limited biodegradability in marine environments, which may constrain the development of specialized degradative communities (Wallbank et al. 2025). Likewise, even when biodegradable substrates initially select for an active biodegrading community—for which the term "*bioplastisphere*" has recently been coined (Zettler et al. 2013)—these communities rapidly transition towards more typical, non-specialized biofilms as maturation progresses (Datta et al. 2016).

Beyond the small fraction of biodegradable plastics, the vast majority of plastics produced globally, and those dominating environmental pollution (Hoseini and Bond 2022), consist of highly recalcitrant polymers, including both chemically inert plastics made of –C–C– polymeric backbones [e.g. polyethylene (PE), polypropylene (PP), polystyrene (PS), and polyvinyl chloride (PVC)] as well as materials such as polyethylene terephthalate (PET) and nylon, which, despite containing hydrolysable bonds, show very limited degradability under natural environmental conditions (Maday et al. 2024, Obrador-Viel et al. 2024). Consequently, most plastics entering the environment behave largely as inert surfaces (Jendrossek 2024). They are therefore colonized by biofilms similar to those forming on any other inert substrate, whether of natural or anthropogenic origin. Nevertheless, substrate-specific selection may still occur under certain conditions, particularly during early colonization stages or at the immediate plastic–microbe interface (Erni-Cassola et al. 2020). However, numerous studies have shown that, at broader community scales, microbial assemblages on plastics often overlap across polymer types and with those developing on inert control materials, with community composition primarily shaped by ecological factors such as location, season, and environmental conditions (e.g. Oberbeckmann et al. 2016, Pinto et al. 2019, Carrillo-Barragán et al. 2025).

Yet, heavily weathered plastics or materials containing non-covalently bound additives (e.g., plasticizers) may transiently support distinctive communities during the earliest stages of colonization (i.e. days) given the leaching of labile substrates (Dussud et al. 2018, Erni-Cassola et al. 2020). Nevertheless, as biofilms mature and leachates are depleted, microbial communities typically converge with those on other inert substrates (Erni-Cassola et al. 2020, Zhang et al. 2024). Future work should therefore investigate plastispheres not only through bulk community analyses but also from a structural, functional, and spatial perspective, focusing on microorganisms directly attached to the plastic surface, where the plastisphere's distinctness may actually reside, rather than the secondary biofilm that later develops. This would extend earlier work by Kirstein et al. (2019), which has received limited follow-up.

These observations suggest that substrate chemistry may influence initial colonization dynamics without necessarily leading to persistent ecological distinctiveness. This raises a broader question: Are plastisphere communities fundamentally different from other biofilms, or do they largely reflect common biofilm assembly processes occurring on persistent synthetic surfaces?

## Plastisphere, a fancy name for a common biofilm?

The first decade of plastisphere research was marked by rapid expansion. The term helped crystallize an interdisciplinary field, bringing together microbial ecologists, polymer scientists, and oceanographers. Importantly, it also shaped how funding agencies and the media engaged with microbial aspects of plastic pollution. In that sense, plastisphere has been more than a descriptor, it has been a catalyst for community building and visibility. Still, the term’s success raises the question of whether its use obscures continuities with broader biofilm research. A fraction of the microbial scientific community questions if it is necessary to create a new category, or could the same research have been contextualized under the long-standing framework of biofilm ecology.

Many traits attributed to the plastisphere are consistent with established biofilm ecology. Recent meta-analyses have also reported statistically detectable effects of plastic substrates on microbial community composition and ecological functions across multiple environments (Li et al. 2024). However, these effects are often context-dependent and coexist with substantial overlap between plastisphere and non-plastic biofilm communities, particularly once broader environmental and successional processes are considered. Surface attachment, extracellular polymeric substance production, and successional dynamics are not unique to plastics (Flemming and Wingender 2010). As mentioned above, comparative studies frequently show substantial taxonomic overlaps between plastic-associated communities and those on inert substrates such as glass, wood, or metal (Pinto et al. 2019, Oberbeckmann et al. 2021), and multi-omic analyses have further demonstrated that environmental context often explains more variation in community composition than polymer type (Oberbeckmann et al. 2016, 2021, Carrillo-Barragán, Cassola and Burkhardt-Holm 2025). Environmental parameters, nutrient regimes, and source communities frequently outweigh substrate chemistry as drivers of microbial assembly. This was beautifully illustrated by the clear differentiation of plastisphere communities between plastics collected in the Atlantic and Pacific Ocean basins (Amaral-Zettler et al. 2015), indicating that plastic-associated microbial communities reflect large-scale environmental gradients and geographic structure rather than surface specificity.

## Ecology of biofilms on plastics, tales of plastispheres from sea and land

Nevertheless, plastics, and their associated biofilms, may differ from most natural particles in two important aspects: (i) their high persistence (Barnes et al. 2009) and (ii) their elevated dispersal (Alimi et al. 2018, Brewer et al. 2020). Never before have such buoyant and highly recalcitrant materials, such as PE or PP, which are less dense than water and can persist for decades, floated in oligotrophic oceans, creating new niches for biofilm development and planetary dispersal. Floating plastics can also support phototrophic colonizers, including algae and cyanobacteria, and recent studies suggest that these organisms may shape plastisphere assembly and local microbial interactions and nutrient dynamics at the air–water interface. Furthermore, in ultraoligotrophic environments, biofilm formation on persistent floating substrates may locally concentrate nutrients and create microscale hotspots of primary productivity, although their broader ecological significance remains uncertain (Rozman et al. 2023, Bairoliya et al. 2024, Binda et al. 2024). Persistence can also extend across environmental compartments. For example, recent work shows that microplastics in lake systems can migrate downward into sediments and groundwater pathways, demonstrating that synthetic polymers may remain in environmental reservoirs for extended periods (Dimante-Deimantovica et al. 2024). This prolonged residence time may allow plastic-associated biofilms to persist far longer than those developing on most natural particulate substrates, potentially altering microbial exposure to environmental conditions across compartments. Hence, although plastisphere research originated in marine systems, plastic contamination in soils is increasingly recognized (Rillig et al. 2023). In soils, plastics are typically embedded rather than freely drifting. Mobility is intermittent and mediated by tillage, erosion, and bioturbation (Brewer et al. 2020). Yet even without continuous movement, persistence remains ecologically significant. Plastics introduce hydrophobic interfaces within mineral matrices and may alter microscale carbon distribution by adsorbing organic matter and xenobiotic compounds, as well as through the release of additives and weathering products. Experimental work indicates that plastic-associated communities in soils may differ from surrounding soil microbiota and, in some contexts, exhibit reduced beta-diversity across sites (Sun et al. 2024). However, the presence of microplastics in soil also interferes with the soil microbiome itself. The material as well as the surrounding biofilm interacts with organic pollutants and interferes with natural soil microbiome functions, such as the nitrogen cycling (Lagos et al. 2025). In soils, therefore, plastisphere distinctiveness arises primarily from microhabitat persistence, interface properties, and interactions with surroundings rather than large-scale dispersal.

Across aquatic and terrestrial systems, the common denominator for plastics is their durability interacting with environmental context. Nevertheless, marine and terrestrial plastispheres differ substantially in dispersal dynamics, physicochemical conditions, and surrounding microbial pools (Brewer et al. 2020, Rillig et al. 2023, Sun et al. 2024). Despite these differences, current evidence suggests that plastic surfaces do not override ecological processes. Rather, they provide persistent, and often mobile, surfaces upon which common microbial assembly mechanisms operate. Beyond microorganisms, plastics might also adsorb and redistribute some hydrophobic contaminants and additives, yet whether they function as important long-range vectors is highly context-dependent and in some settings probably modest (Yu et al. 2021, Rafa et al. 2024).

While the previous sections focus on substrate properties and community assembly processes, claims of plastisphere distinctiveness have also centered on function. In particular, biodegradation and enrichment of pathogens and antimicrobial resistance genes have been repeatedly called for as defining features of plastic-associated biofilms (Wu et al. 2019, Yang et al. 2019, Amaral-Zettler et al. 2020, Wright et al. 2020). These narratives position the plastisphere either as a potential reservoir of biotechnological innovation or as a vector for ecological and public health risk. The question, therefore, is not only whether plastisphere communities are compositionally distinct but whether they are functionally enriched in ways that justify treating them as a separate ecological entity.

For most persistent plastics, ecological consequences arise less from assimilation and more from extended residence time. Plastics remain where natural organic particles would decay, providing stable surfaces across successional timescales that exceed those of biodegradable substrates (Vass et al. 2024).

## Plastic biodegradation, a feature used as biofilm uniqueness with limited evidence

Beyond questions of community assembly, plastisphere distinctiveness has often been inferred from presumed functional capacities, particularly plastic biodegradation. Hydrocarbon biodegradation, such as the presence of hydrocarbonoclastic bacteria or genes encoding classical hydrocarbon-degrading enzymes like alkane monooxygenases (*alkB*), has frequently been used as a proxy for plastic degradation. The detection of these elements has often been used to support the uniqueness of the plastisphere and to suggest that plastic biodegradation occurs under environmental conditions. However, there is limited direct evidence for plastic biodegradation in natural environments due to the inherent recalcitrance of these materials (Oberbeckmann and Labrenz 2020) and the difficulty of implementing robust methods capable of unequivocally monitoring such slow processes (Obrador-Viel et al. 2024). Considering soils as potential environmental hotspots for plastic biodegradation (Contreras-Moll et al. 2025), even terrestrial soil burial studies based on gravimetric weight-loss measurements have generally reported very limited degradation of conventional plastics such as PE, whereas substantial deterioration is more consistently observed for biodegradable agricultural mulches and other designed biodegradable polymers (Sintim and Flury 2017, La Mantia et al. 2020). Nevertheless, these approaches often struggle to disentangle abiotic fragmentation, additive loss, surface erosion, and true microbial mineralization of polymer carbon, highlighting the methodological limitations associated with interpreting weight loss as unequivocal evidence of biodegradation (Sander 2019, Francioni et al. 2022). Consequently, some authors have questioned whether meaningful plastic biodegradation occurs at all under environmental conditions (Jendrossek 2024). At the same time, several studies using isotopically labeled or heavily weathered plastics have demonstrated partial mineralization and assimilation under specific experimental conditions (Zadjelovic et al. 2022, Vaksmaa et al. 2023). These findings indicate that some degree of microbial utilization of plastic-derived carbon can occur, particularly after abiotic weathering, oxidative pre-treatment, or prolonged environmental ageing, which may generate lower-molecular-weight oxidation products that are more bioavailable to microorganisms. However, these observations also raise important conceptual questions regarding what should be considered true plastic biodegradation, as microorganisms may preferentially assimilate oxidized fragments, additives, or low-molecular-weight compounds generated during weathering rather than intact polymer chains themselves. Consequently, while these studies provide important mechanistic evidence that microbial assimilation of plastic-derived carbon is possible under certain conditions, the ecological relevance, environmental rates, and extent to which such processes contribute to meaningful degradation of persistent plastics in natural systems remain uncertain.

Importantly, the environmental context in which plastisphere communities develop differs substantially between marine and terrestrial systems, with important implications for the potential occurrence of biodegradation. Marine environments are often oligotrophic and characterized by low nutrient availability, lower temperatures, and limited retention of degradative enzymes near the polymer surface, conditions that may further constrain microbial utilization of highly recalcitrant plastics (Oberbeckmann and Labrenz 2020, Wright et al. 2020). In contrast, soils harbor far greater microbial diversity, physicochemical heterogeneity, and prolonged particle residence times, potentially increasing opportunities for plastic–microbe interactions and oxidative weathering processes (Rillig et al. 2023). Consistent with this, plastic biodegradation rates reported in marine systems are generally lower than those observed in terrestrial environments (Delacuvellerie et al. 2019, Marín et al. 2025), and comparative analyses have suggested orders-of-magnitude lower biodegradation potential in marine relative to terrestrial microbiomes (Contreras-Moll et al. 2025). Nevertheless, even in terrestrial systems, convincing evidence for substantial biodegradation of persistent polyolefins under environmentally relevant conditions remains scarce (Jendrossek 2024). Together, these differences further suggest that putative biodegradation processes are highly context-dependent rather than a universal defining feature of plastisphere communities. Plastics are essentially very high-molecular-weight hydrocarbons, and therefore an enrichment of typical hydrocarbon-degrading microbes within the plastisphere would not be unexpected if substantial amounts of substrate were indeed being degraded. However, to the best of our knowledge, no study has simultaneously demonstrated (i) the enrichment of putative hydrocarbon-degrading microorganisms on plastics relative to inert surfaces (e.g. glass or ceramics) and (ii) *in situ* polymer breakdown and assimilation by these organisms. More concerning is how little we still know about the enzymes required for the initial scission of recalcitrant polymer chains and the metabolic pathways involved in the assimilation of the resulting breakdown intermediates (Wright et al. 2020). While enzymatic degradation has been demonstrated for certain hydrolyzable polymers such as PET (Yoshida et al. 2016), and PET hydrolases, although rare, have been detected in marine and terrestrial metagenomes (Danso et al. 2018, Alam et al. 2025), enzymes capable of attacking polymers with recalcitrant –C–C– backbones remain largely elusive. Research has begun to provide evidence for mechanistic assimilation of polyethylene degradation products (Gravouil et al. 2017, Zadjelovic et al. 2022), intriguingly, suggesting that alkane monooxygenases (*alkB*), commonly used as biomarkers for PE biodegradation in plastisphere metagenomes, may not even be required. Instead, polymer scission likely involves strong oxidative processes that generate a high diversity of oxidized aliphatic compounds rather than alkanes (Obrador-Viel et al. 2025).

However, the lack of clear evidence for plastic biodegradation does not necessarily imply that hydrocarbon-degrading microbes are irrelevant in plastisphere communities or that they cannot serve as distinctive biomarkers within these biofilms. In fact, an enrichment of hydrocarbon-degrading taxa (Erni-Cassola et al. 2020) and β-oxidation pathways, key for PE assimilation (Obrador-Viel et al. 2025), has been observed during the very early colonization stages of PE in marine environments. Similarly, early-stage biofilms on PP in Antarctic waters also displayed a marked presence of hydrocarbon degraders, with *Oleispira* emerging as the second most dominant taxon (Monràs-Riera et al. 2024). Nevertheless, these initial enrichments appear to be transient and are rapidly replaced over time by more typical biofilm communities that resemble those developing on inert surfaces (Wright et al. 2019). This pattern suggests that widespread biodegradation of durable polyolefins is unlikely to represent a dominant ecological process within mature plastisphere communities (MacLeod et al. 2021). Consequently, future studies should incorporate structural and spatial analyses of plastisphere biofilms, as plastic-degrading microorganisms are likely to persist only as minor populations at the plastic surface, potentially masked by the bulk mature biofilm that develops over time.

## Biofilms, and not only plastispheres, are hotspots of antibiotic resistance genes and potential pathogens

Biofilms are widely recognized as hotspots for antibiotic resistance genes (ARGs). This is expected, as biofilms consist of dense microbial communities in which cells interact closely, engaging in both cooperation and competition. Their high cell density and spatial proximity promote horizontal gene transfer, though mechanisms such as conjugation, transformation, and phage-mediated transduction (Sørensen et al. 2005) and plastics are no exception (Zhang et al. 2020). Consequently, biofilms provide an optimal environment for the exchange and expression of genetic traits related to both antibiotic production and antibiotic resistance.

However, whether plastisphere communities harbor more ARGs than other types of biofilms remains unclear. Each biofilm develops from a specific source community, forms on a particular surface, and is shaped by its physicochemical and environmental context. These factors drive distinct ecological colonization processes and result in considerable variability in bacterial community structure and, consequently, in ARG composition. For example, Oberbeckmann et al. (2021) reported a higher abundance of ARGs on wood surfaces than on plastics. Similarly, Zadjelovic et al. (2023) reported similar abundances and compositions of ARGs on wood and pristine PE, with slightly higher levels on weathered PE, while the overall biofilm repertoire differed markedly from that found in the planktonic fraction. Wu et al. (2019) found similar ARG diversity in biofilms on rock and plastic, with slightly higher richness in leaf-associated biofilms, although notable differences were still present. Interpretation of these data is challenging because multiple factors beyond polymer type can influence bacterial recruitments and microbial interactions, including the production and dissemination of ARGs. Such factors include hydrophobicity, aging state, and substrate biodegradability (Yang et al. 2025, Huang et al. 2026).

Concerns about pathogen enrichment on plastics emerged early in plastisphere research. The original study introducing the concept reported high relative abundances of *Vibrio* sequences in one plastic sample, raising concerns that plastics might act as vectors for pathogenic microorganisms (Zettler et al. 2013). Subsequent studies have similarly detected bacterial taxa, including *Vibrio, Pseudomonas, Arcobacter*, and *Aeromonas* on environmental plastics (Amaral-Zettler et al. 2020, Wright et al. 2020). However, most plastisphere studies rely on short-read amplicon sequencing, which typically lacks the taxonomic resolution necessary to determine whether detected taxa correspond to pathogenic strains. Genera such as *Vibrio* or *Pseudomonas* are widespread primary colonizers of natural surfaces and common members of environmental biofilms, and their presence therefore does not necessarily imply pathogenicity. Comparable questions regarding surface selectivity have also been proposed for fungal colonizers of plastics, although these remain insufficiently resolved (Gkoutselis et al. 2021, 2024). More broadly, biofilms themselves, not only plastispheres, can concentrate opportunistic microorganisms simply due to their high cell densities and favorable growth conditions, offering protection under harsh environments where these would normally be outcompeted. While some studies have detected microorganisms with potential pathogenicity on environmental plastics, these observations frequently lack adequate controls, such as comparisons with natural substrates or experimental infection assays to confirm pathogenicity (Beloe et al. 2022, Metcalf et al. 2022). Those studies that did include inert surface controls reported similar taxa than biofilms developing on natural particles, wood, algae, or mineral substrates (Oberbeckmann et al. 2016, Amaral-Zettler et al. 2020). If plastics truly enriched specific pathogens, what biological or ecological mechanism would account for such selective enrichment? In addition, several studies have reported a rapid decline of initial colonizing pathogens as plastisphere communities transition into riverine and marine environments, consistent with the strong ecological turnover observed for the broader microbial community (Ormsby et al. 2023, Tulloch et al. 2024, Stevenson et al. 2025). Thus, although plastics may occasionally transport microorganisms originating from contaminated environments, the available evidence suggests that pathogen enrichment on plastics largely reflects the general ecology of biofilms and their environmental context rather than a unique property of plastic surfaces.

What appears consistent across studies is that both ARG and potential microbial pathogen abundance are generally higher in biofilms than in planktonic communities (Yang et al. 2019, Liang et al. 2023, Zadjelovic et al. 2023). However, there is little evidence that plastics themselves act as hotspots of such accumulation when compared with other co-occurring inert materials, with the surrounding environment again emerging as the primary driver of common biofilm development, regardless of substrate type. Nevertheless, given the persistence and high dispersal of plastic materials, plastispheres may contribute to the long-term stability, accumulation, and spread of ARGs and pathogenic microbes in the environment. This may occur, for instance, when plastics escape conventional wastewater treatment plant (WWTP) retention, transporting plastisphere communities formed in environments with high ARG and pathogen loads into natural ecosystems. At the same time, available evidence indicates that these communities undergo rapid ecological turnover once released into natural environments. Studies have shown minimal differences between plastics and control surfaces, alongside strong shifts in microbial community composition as biofilms move from WWTP systems into freshwater rivers and eventually marine environments. During this transition, the original WWTP-associated microbiome undergoes rapid ecological turnover and becomes progressively dominated by naturally occurring microorganisms adapted to the receiving environment (Ballesté et al. 2024, Tulloch et al. 2024, Stevenson et al. 2025). Nevertheless, some studies have reported intriguing enrichments of some specific ARGs or pathogens on plastics, highlighting the need for further work to elucidate the mechanisms that may drive these patterns (Zadjelovic et al. 2023, Stevenson et al. 2025).

## Methodological lenses and biases

The perceived uniqueness of plastisphere communities is also shaped by the methods used to study it. Polymer type, incubation duration, sequencing resolution, and bioinformatics pipelines can all exaggerate or downplay differences. For example, a number of recent studies have highlighted that plastisphere patterns are more dependent on experimental framing than on substrate chemistry alone, with standardized surveys across multiple Mediterranean and Atlantic locations showing community composition tracking geography, exposure time, and environmental variables more consistently than polymer identity (Lacerda et al. 2025); time-series incubations demonstrating that early-stage differences among plastics often reflect opportunistic colonizers such as *Alteromonas* and *Vibrio*, whereas longer deployments reveal increasing similarity to surrounding seawater communities (Lemonnier et al. 2022); field comparisons between oceanic regions indicating environmental exposure and incubation duration outweigh plastic type in structuring assemblages (Dudek and Neuer 2023); and successional trajectories in subtropical coastal waters showing convergence across materials over weeks to months (Xu et al. 2025). Even year-long wastewater incubations report chaotic dynamics and limited evidence for a stable, substrate-specific plastisphere (Tagg et al. 2022).

More broadly, biogeography has been shown to dominate colonization dynamics across plastic types (Coons et al. 2021), and mature biofilms on synthetic polymers frequently share a general marine biofilm core rather than forming polymer-exclusive communities (Kirstein et al. 2018). Together, these studies highlight that apparent plastisphere uniqueness is often dependent on spatial scale, incubation length, and analytical resolution, reinforcing the need to distinguish ecological signal from methodological bias.

## Persistence and mobility: the differentiating factors

Therefore, if plastic biofilms are not consistently taxonomically distinct, and if degradation is often minimal, what then justifies retaining a separate term? The answer, in our view, lies in the peculiar combination of durability and, particularly in aquatic systems, movement. PE, PP, and PS persist for decades. Being less dense than water, their buoyancy allows them to move downstream through river networks and drift across ocean basins, or even be transported atmospherically across continents (Kubowicz and Booth 2017, Coons et al. 2021). Unlike most natural organic substrates such as leaves, wood, or marine snow, which are transient and rapidly decay, plastics can persist for decades across environmental compartments. Abiotic particles such as mineral dust, sediments, and sand can likewise be mobilized by winds and water flow and transported over long distances; however, their substantially higher densities generally reduce buoyancy, floating persistence, and long-range dispersal potential relative to many common plastics. Hence, plastics combine persistence and mobility at unprecedented global scales (Griffin 2007, Abbasi et al. 2022).

A persistent, mobile substrate changes the geometry of dispersal. Microbes attached to plastics are not merely colonizing a surface but they are travelling (Zhao et al. 2025). Plastics connect wastewater effluents, rivers, sediments, estuaries, coastal zones, and the open ocean. They link anthropogenic and natural systems in ways that biodegradable materials rarely do. They have the potential to connect habitats and their longevity allows the microplastics and the microbiome attached to them to travel from rivers and shores to the deep sea (Cutroneo et al. 2022). Microplastics and with them the biofilm have the potential to accumulate and persist in the sediments, reaching deep layers, which are largely inaccessible to natural materials.

Dispersal is a fundamental process in microbial biogeography (Hanson et al. 2012). While other natural and anthropogenic particles, including mineral dust, sediments, glass, and stone, may also transport microorganisms, plastics introduce a distinct class of vectors: long-lived, synthetic, low-density materials that are globally distributed and continuously accumulating. Thus, even if the microbial taxa involved are not themselves unique, the spatial and temporal dynamics generated by these highly persistent and mobile substrates may be. In this sense, the plastisphere becomes scientifically meaningful not because plastics necessarily host unprecedented microbial communities, but because they create novel dispersal infrastructures connecting ecosystems across large spatial and temporal scales.

## Communication, policy, and disciplinary identity

Beyond these ecological considerations, the term plastisphere has also shaped how microbial perspectives of plastic pollution are communicated. Even if plastic-associated communities are not biologically unique in the strict sense, the term has communicative power. It provides a concise way to capture the idea of microbial life structured by persistent synthetic substrates, making complex ecological processes accessible to policymakers, NGOs, and the public. In that respect, plastisphere has functioned not only as a scientific concept but also as a narrative device that has made available microbial ecology within broader environmental debates.

## Conclusion

Bulk plastic-associated microbial communities share many features with classical biofilms that may form on any other inert surface. They are shaped by environmental context, governed by familiar successional processes, and rarely demonstrate unequivocal large-scale polymer mineralization, with any potential plastic–microbe interface distinctions often obscured beneath layers of common biofilm growth. Although marine and terrestrial plastispheres differ substantially in physicochemical conditions, dispersal dynamics and microbial source pools, current evidence suggests that the same overarching ecological principles govern community assembly across both systems, with environmental context generally outweighing polymer identity as a driver of mature biofilm structure. Yet, plastics have introduced a combination of longevity and mobility unlike most natural substrates. They create durable, traveling microhabitats that connect ecosystems across space and time. This infrastructural novelty, rather than taxonomic uniqueness, is what justifies retaining the term. Plastisphere should therefore not be called for as evidence of ecosystem uniqueness. It should denote a specific ecological condition of the Anthropocene: microbial life colonizing persistent, mobile, synthetic surfaces that reshape dispersal and residence time. Used with that precision, the term remains conceptually useful.

This piece is not intended to settle the debate but to contribute a rational point of view and to provoke reflection. We welcome input from the community on whether 'plastisphere' remains a useful scientific concept or whether it is time to fold it back into the broader umbrella of biofilm research.
